# Performance of contrast enhanced SSFP and T2-weighted imaging for determining myocardium at risk in a multi-vendor, multi-center setting- data from the MITOCARE and CHILL-MI trials

**DOI:** 10.1186/1532-429X-17-S1-P194

**Published:** 2015-02-03

**Authors:** David Nordlund, Gert Klug, Einar Heiberg, Sasha Koul, Terje H Larsen, Bernhard Metzler, David Erlinge, Dan Atar, Marcus Carlsson, Henrik Engblom, Håkan Arheden

**Affiliations:** Cardiac MR group Lund, Dept. of Clinical Physiology, Lund University, Lund, Sweden; Dept. of Cardiology, Medical University of Innsbruck, Innsbruck, Austria; Dept. of Cardiology, Lund University, Lund, Sweden; Dept. of Cardiology B, Oslo University Hospital Ullevål, and Faculty of Medicine, University of Oslo, Oslo, Norway; Dept. of Heart Disease, Haukeland University Hospital, Bergen, Norway

## Background

Myocardial salvage, as determined by cardiac magnetic resonance imaging (CMR), is increasingly used as an endpoint in clinical trials. In order to calculate myocardial salvage, the infarct size needs to be related to myocardium at risk (MaR). MaR has previously been assessed by both T2-weighted imaging and contrast enhanced SSFP (CE-SSFP). The aim of this study was to determine how T2-weighted triple inversion recovery imaging (T2w) and CE-SSFP perform in determining MaR in multi-vendor, multi-center clinical cardioprotection trials.

## Methods

All patients who underwent CMR in the recently published MITOCARE and CHILL-MI trials were included, comprising 212 patients from 17 different sites in six countries. All patients underwent primary PCI for acute ST-elevation myocardial infarction and CMR was performed within 2-6 days. Late gadolinium enhancement, T2w and CE-SSFP images were acquired on a GE, Philips or Siemens 1.5T system. Myocardium at risk, diagnostic quality and culprit vessel (later compared to angiography) was manually evaluated by expert observers blinded to all other data. If an observer was able to identify and define MaR, the images were considered diagnostic.

## Results

In 97.2% of patients the CE-SSFP was considered diagnostic versus 69.8% for T2w, with higher inter-site variability for T2w images. Culprit vessel was correctly identified in 96.6 % of all patients using CE-SSFP versus 88.8% using T2w (p=0.0032). Myocardium at risk did not differ significantly as measured by CE-SSFP or T2w in diagnostic images (LAD: 44±10% versus 44±9%, p-value: 0.78, LCx: 30±9% versus 30±12%, p-value: 0.96, RCA: 31±7% versus 30±8%, p-value: 0.44). When comparing the three different vendors one was found to have a significantly higher degree of diagnostic T2w images (88.1%) than the other two vendors (56.1% and 63.3%, p<0.0001 and p=0.0051) while the degree of diagnostic CE-SSFP images was similar for all three vendors (96.4%, 96.9% and 100%, p-value: 1.0, 0.57 and 1.0).

## Conclusions

In diagnostic images T2w showed similar results to CE-SSFP. However, CE-SSFP had a consistently higher degree of diagnostic images across vendors and sites. If the MITOCARE and CHILL-MI trials had relied on T2w for MaR assessment, 30% of the patients would have been excluded from CMR analysis, increasing the number of patients needed to complete the trials.

**Figure 1:**
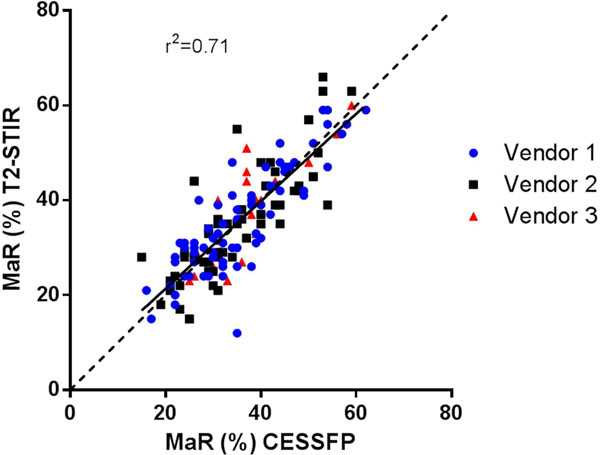
MaR as measured by CESSFP vs T2w using only datasets diagnostic by both methods (143/212) and grouped by vendor.

**Figure 2:**
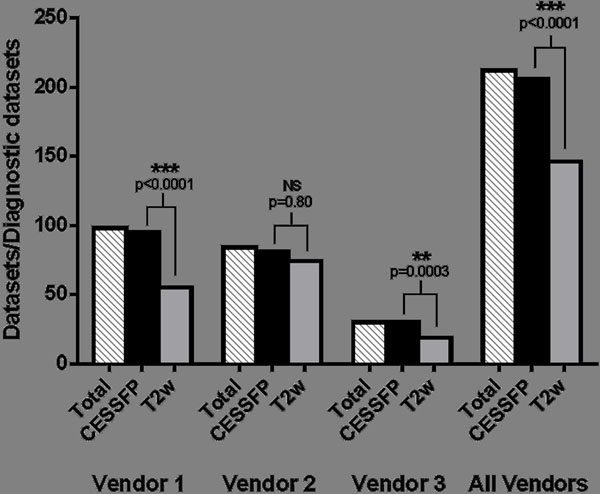
Number of diagnostic datasets by CESSFP and T2w compared to the total number of patients, grouped by vendor and altogether, respectively.
